# Who joins voluntary conservation programs? Socioenvironmental predictors of participation in tropical working landscapes

**DOI:** 10.1007/s13280-026-02381-3

**Published:** 2026-04-04

**Authors:** Ramon Felipe Bicudo da Silva, Felipe Altivo, Juliana Angelo, Denise Silva Leão de Souza, Henrique Simões Carvalho Costa, James D. A. Millington, Andrés Viña

**Affiliations:** 1 Laboratory of Spatial Studies, Environmental Conservation and Sustainability (LAECAS), Center for Environmental Studies and Research (NEPAM), State University of Campinas (UNICAMP), Campinas, 13083-870 Brazil; 2 Department of Geography, King’s College London, London, WC2R 2LS UK; 3 Center for Systems Integration and Sustainability, Department of Fisheries and Wildlife, Michigan State University, East Lansing, MI 48823 USA

**Keywords:** Atlantic forest, Bayesian INLA model, Environmental policy, Policy assessment, Sustainability, Working landscapes

## Abstract

Voluntary conservation programs are increasingly used to align rural development with ecosystem protection within working landscapes, yet their effectiveness depends on the willingness and capacity of landowners to participate. To explore factors that influence participation, we investigated socioenvironmental characteristics of rural properties in a conservation program in the Brazilian Atlantic Forest. Using Bayesian modeling across 18 717 properties, we found participants concentrated in marginal agricultural lands and in properties with large extents of riparian areas, suggesting a pattern of self-selection into program participation. Using participation probabilities, we identified ~ 5000 additional properties to join future conservation programs, in the same area. These findings suggest that incentive-based policies may reward voluntary conservation while successfully engaging landowners in marginal lands. Voluntary conservation schemes tend to attract landowners predisposed to conservation, while struggling to engage those facing higher opportunity costs. Achieving transformative outcomes will require incentive structures that better align economic competitiveness with ecological priorities.

**Supplementary Information:**

The online version contains supplementary material available at https://doi.org/10.1007/s13280-026-02381-3.

## Introduction

Reconciling environmental conservation with economic development in human-modified working landscapes has remained a persistent global challenge over recent decades (Garibaldi et al. 2020; Duarte et al. 2025). A variety of strategies have been put in place across scales (from local to global) to address this issue, including the establishment of sustainable production standards assessed through eco-certification systems (Silva et al. 2019), supply chain agreements (Dou et al. 2018), and by the enforcement of legal mechanisms to limit violations against environmental legislations, such as illegal hunting or logging (Merkus 2024; Nunes et al. 2024). In general, “stick” strategies established under punitive frameworks that seek to punish offenders (Silva et al. 2024) may work in some contexts but fail in others (Dou et al. 2018). In conservation policy, ‘sticks’ typically include regulatory tools such as fines, sanctions, embargoes, or mandatory restoration orders. Conversely, “carrot” conservation strategies may offer positive incentives to entice individuals to participate in conservation programs (Silva et al. 2024).

In such “carrot” approaches, individuals (hereinafter ‘rural landowners’) are incentivized to voluntarily participate in conservation programs that include not only sustainable production schemes within agricultural systems but also natural conservation and ecosystem restoration (Viani et al. 2019; Silva et al. 2025a). Programs of payment for ecosystem services (PES) are strategies capable of incentivizing rural landowner’s participation in conservation efforts without compromising their economic outcomes (Wunder 2005; Silva et al. 2017).

An important dimension in aligning production and conservation goals at the farm or landscape scale is the cost associated with adopting new management practices or reorganizing production (Sainz-Santamaria 2024). Evidence shows that even when rural landowners are willing to implement sustainable practices, such as restoring native vegetation, high and non-affordable costs often act as a barrier to participation (Wunder 2007; Brancalion et al. 2019). In this context, economic incentives and conservation tools become critical to lower these barriers and enable broader engagement of landowners (Selinske et al. 2016). However, voluntary incentive-based conservation schemes (e.g., PES) may sometimes create an ‘enrollment bias’, where individuals already inclined toward conservation are favored over those whose activities have a potential higher negative environmental impact on the environment (e.g., higher deforestation risk), thereby reducing the overall effectiveness of such strategies (Robalino and Pfaff 2013).

Over the last two decades, dozens of PES programs have emerged in Brazil (Salzman et al. 2018; Mota et al. 2023), but the effectiveness of many of them has not been properly evaluated (Mota et al. 2023; Silva et al. 2025a). The Atlantic Forest biome, one of the most globally endangered tropical ecosystems (Solórzano et al. 2021), has been the focus of several local to regional conservation initiatives. One such initiative is the *Projeto Conexão Mata Atlântica* (PCMA), implemented in the working landscapes of Paraíba and Ribeira Valleys of state of São Paulo. These working landscapes are human-dominated production mosaics located outside protected areas and are used for farming, ranching, and forestry (Killion et al 2018; Garibaldi et al. 2020). Besides this study follows the working landscape definition of Garibaldi et al. (2020), we note the use of working landscape to refer urbanized or industrial settings with managed green spaces (Antrop 2004). As a regional initiative aimed at creating knowledge and practice for scaling up conservation practice, in-depth analyses of PCAM enable acquiring insights that are applicable at state and national scales.

As a premise, this study evaluates the likelihood of participation in the PCMA based on the rural property’s size, location/accessibility, topography, compliance level with the national Forest Code (Brazilian Law 12651 of 2012), water resources, conservation status, and climate regimes. Therefore, the study empirically evaluates the degree to which those socioenvironmental predictors contribute to explaining the participation of rural landowners in the PCMA, while looking for the presence of enrollment bias. Such knowledge is crucial for leveraging future scaling up of State level policies aimed at reconciling agricultural development with ecosystem conservation and restoration within working landscapes. In addition, we use this regional case to derive generalizable insights for incentive-based conservation in the Atlantic Forest and other biodiverse, economically strategic regions. Considering the Atlantic Forest loss of 16 Mha of native vegetation over the past four decades (Silva et al. 2025b), primarily due to agricultural expansion (Silva et al. 2023), conservation strategies should prevent enrollment bias by targeting productive agricultural lands typically linked to lower conservation value and higher degradation risks.

The study aims to identify which property-level attributes are most strongly associated with voluntary engagement in a large-scale, incentive-based conservation program. Specifically, we seek to identify which socioenvironmental characteristics of rural properties explain voluntary participation in the *Projeto Conexão Mata Atlântica* (PCMA) in São Paulo State, by addressing the following questions: (*i*) To what extent do property-level features (e.g., topography) influence the likelihood of landowner participation in the PCMA? (*ii*) Does participation reflect an enrollment bias toward marginal agricultural lands (e.g., low profitability, high ecological value), or are agriculturally productive lands equally likely to engage?

## Materials and methods

### Case study

**Fig. 1:**
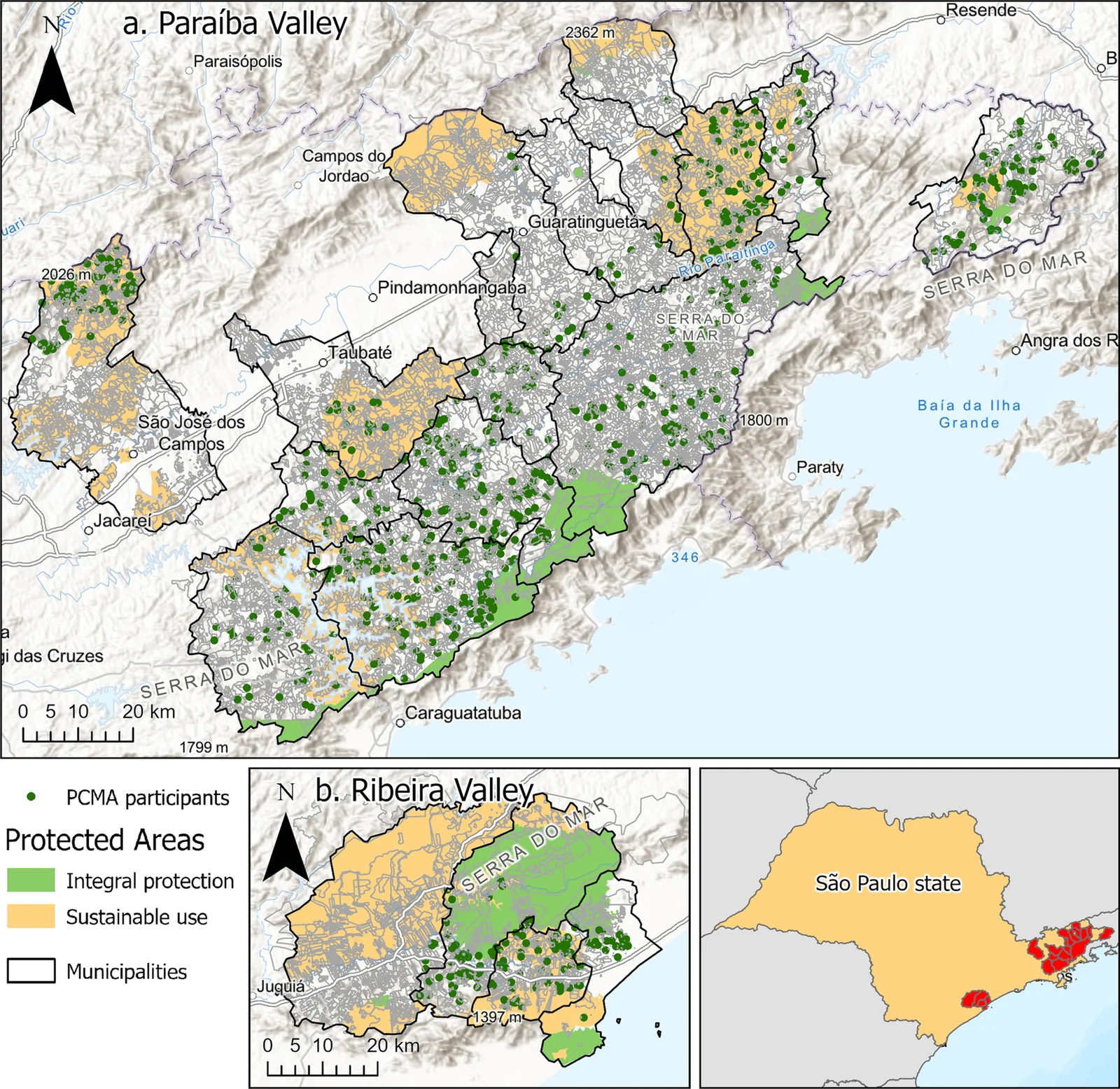
São Paulo state’s areas targeted by the Projeto Conexão Mata Atlântica (PCMA), a conservation program. **a** The Paraíba Valley region with around 800 PCMA participants. **b** The Ribeira Valley, with about 150 PCMA participants

The *Projeto Conexão Mata Atlântica* (PCMA) was a pilot conservation program led by the Brazilian Ministry of Science and Technology with financial support from the Global Environmental Facility (Oliveira et al. 2024). The program was implemented in three states—Minas Gerais, Rio de Janeiro, and São Paulo. As a pilot initiative spanning three states, the PCMA supported various conservation strategies, but only São Paulo translated these efforts into a large-scale PES program. In São Paulo, the institution responsible for its execution was the *State Secretariat for Environment, Infrastructure and Logistics* (SEMIL) (Silva et al. 2025a). Hence, the PCMA in São Paulo state involved over a thousand participants within 20 municipalities in Paraíba and Ribeira valleys (Fig. 1), during 2018–2024. The PCMA was developed through a co-production approach, involving the participation of public and private agencies, civil organizations, and landowners’ representatives (Pereira 2024). To address a variety of landowner types and socioeconomic contexts (Oliveira et al. 2024), the program provided support through four categories of payment schemes: multiple-use (sustainable agriculture), protection (NVC conservation and restoration), certification (eco-label assistance), and sustainable value chains (agroforestry development). Given the PCMA’s positive outcomes at the rural property level, such as additional gains of 1 ha on average per participant property, and improved pastureland quality (Silva et al. 2025a), we argue that identifying which socioenvironmental predictors of rural properties are most strongly associated with participation provides essential insights for informing policy design at both the state and biome scales. Therefore, we use our results to predict the likelihood of new landowner participants if the program scales into a state-level policy—by explicitly mapping potential participation and calculating the budgetary impact considering the current values spent by the PCMA.

### Datasets and data preparation

We combined multiple publicly available spatial datasets to derive property-level indicators of land-use, environment, and accessibility across São Paulo State, Brazil. Native vegetation and pasture quality were obtained from MapBiomas (2023a, 2023b), and topographic data from INPE/TOPODATA (Valeriano 2008). Climate data (mean annual precipitation, 1970–2000) was derived from WorldClim v2.1, while transportation costs from Victoria et al. (2021). Legal and cadastral boundaries of private rural properties were sourced from SEMIL for PCMA participants, from the Rural Environmental Cadastre (CAR) for non-participants, and public protected area polygons from the São Paulo State Environmental Spatial Data Infrastructure (DataGEO). For modeling purposes, rural property shapefiles were joined into a single file in which participant properties were identified with a value of 1, while non-participant properties with a value of 0.

All datasets were harmonized at the rural property level (which constitutes the unit of analysis in the study) using zonal statistics. For each property, we computed the percentage of native vegetation, mean pasture quality, elevation, slope, aspect, and accessibility (transportation costs), along with distance to protected areas. Variables related to legal constraints—such as property size (in fiscal modules, FM) and the extent of Permanent Preservation Area (APP), which are riparian buffer zones that should be preserved under the Forest Code. Thus, we adopted the riparian APP extent as variable to indicate presence of water resources within the property. Based on the riparian APP and native vegetation data, we calculate the deficit of vegetation cover within those areas. Properties ≤ 0.5 ha were excluded to avoid geometric artifacts, as suggested by Pienkowski et al. (2025). Full details on data sources, processing workflow, and variable definitions are provided in Supplementary Material—SM Dataset and SM Data preparation.

### Rationale of the explanatory variables

Recent studies in Brazil (Fernandes et al. 2025) and in international contexts (Kline et al. 2000) have demonstrated that rural landowners are more likely to restore native vegetation to protect water resources. Property size is not conclusive as an indicator of the willingness for restoration in Brazilian contexts (Tavares et al. 2024; Nardy et al. 2025), neither a PCMA requirement conditioning participation (Silva et al. 2025a), but an influencing factor according to studies in Central and North America (Lubell et al. 2013; Duke et al. 2014). In addition, non-compliance with the Forest Code (a situation when a rural property does not have the minimum native vegetation cover required by Law) may motivate landowners to participate in such initiatives to receive financial support and technical assistance for meeting legal requirements—such as restoring at least 20% of their properties with native vegetation (Brancalion et al. 2016). The location of a rural property is an important determinant of its competitiveness, as those closer to consumption and logistics hubs are more economically advantageous (Pienkowski et al. 2025). Such economically favorable location of a rural property may act negatively to the participant’s willingness to engage in similar conservation initiatives, as was observed in Ecuador (Mohebalian and Aguilar 2016). We expect steep and south-facing slopes to represent a disadvantage for agricultural activities (Borda-Ninõ et al. 2021), constituting high vulnerable areas for intense erosion processes, thus favoring participation in conservation programs (Liu et al. 2008; Duke et al. 2014). Table 1 summarizes the socioenvironmental variables considered in our model and the theoretical or empirical rationale for their inclusion, as supported by previous studies. Rather than assuming the direction of the association a priori, our goal was to test whether these variables influence landowner participation in the PCMA. If participation is largely driven by marginal agricultural properties already prone to conservation (e.g., highest proportions of native vegetation), this indicates an enrollment bias in the PCMA (Robalino and Pfaff 2013).

**Table 1:** Socioenvironmental variables included in the models and the theoretical or empirical rationale for their selection, based on previous studies

Variable	Rationale	References
Native vegetation (2017)	Producers with greater amounts of native vegetation are more likely to seek participation to gain benefits or rewards associated with their property's conservation status	Nielsen et al. (2018), Viani et al. (2019)
Riparian permanent preservation area	The presence of water resources on a property is a stronger incentive for landowners to participate in water conservation initiatives	Kline et al. (2000), Fernandes et al. (2025)
Deficit of riparian permanent preservation area	Landowners may view conservation programs as an opportunity to restore vegetation in APPs and comply with legislation at no personal cost	Oliveira et al. (2024)
Transportation cost (2017)	Properties farther from consumption centers or with poor road access may be less economically competitive, increasing the likelihood of participating in conservation programs for additional benefits	Mohebalian and Aguilar (2016), Silva et al. (2025a)
Rural property size	Larger properties offer more flexibility to adopt conservation practices without disrupting existing land uses, while smaller properties face fewer legal restrictions and thus have fewer incentives to participate	Lubell et al. (2013), Duke et al. (2014), Tavares et al. (2024), Nardy et al. (2025)
Elevation	High-elevation areas often face greater land-use and settlement restrictions, which may encourage landowners to participate in conservation programs	Silva et al. (2020)
Slope	Steep-slope areas often face greater land-use and settlement restrictions, induce intense erosion processes, which may encourage landowners to participate in conservation programs	Liu et al. (2008), Duke et al. (2014), Viani et al. (2019), Lemos et al. (2021)
Aspect	South-facing slopes are less suitable for agriculture, which may encourage landowners with predominantly south-oriented terrain to pursue alternative rural activities, including conservation efforts	Silva et al. (2016)
Pasture quality (2017)	Properties with highly degraded resources, such as low-quality pasture, may motivate landowners to adopt agricultural-management practices to restore land quality and improve agricultural productivity	Silva et al. (2025a)
Protected area distance	Proximity to or inclusion in protected areas often entails stricter land-use regulations, making landowners more likely to engage in conservation due to oversight and opportunities for public support	Oliveira et al. (2024), Godoy et al. (2024)
Precipitation	In the study region, areas with high precipitation—typically at higher elevations and with greater forest cover—may be more likely to engage in conservation, as landowners may view these areas as key to providing water-related ecosystem services	Lemos et al. (2021)

### Statistical analysis

This study applied the Integrated Nested Laplace Approximation (INLA; Martins et al. 2013) to fit a spatially explicit generalized linear mixed model (GLMM) and assess the effects of multiple predictors on the probability of ‘landowner's participation in the PCMA’ (response variable). All analyses were conducted in R using the INLA package (Rue et al. 2009). The response variable was modeled using a binomial distribution with a logit link function.

INLA is a fast Bayesian framework for latent Gaussian models that uses integrated nested Laplace approximations, rather than Markov Chain Monte Carlo (MCMC) (Moraga 2019). It fits the same binomial–logit likelihood as a standard GLM, but allows latent components (e.g., random effects—including *spatially structured* effects—and nonlinear smoothers) and returns full posterior parameter uncertainty. This is particularly useful because a standard binomial GLM assumes independent observations and estimates only fixed effects. Here we use INLA to handle the multilevel structure (to account for variation in engagement with the PCMA across municipalities) by including a municipality-level random intercept (*independent random noise* in the INLA documentation) as an independent and identically distributed effect, which accounts for clustering of properties within municipalities, thereby addressing the resulting non-independence among observations and extra-binomial variation (Blangiardo and Cameletti 2015).

Following a previous approach (Pienkowski et al. 2025), all numerical variables were scaled to have mean of zero and variance of one. Standardizing predictors prior to analysis is widely recommended in Bayesian modeling, as it simplifies the specification of priors and often improves numerical stability of the estimation (McElreath 2020). Standardization is particularly important when variables are measured on very different scales or have skewed distributions, as it makes predictor values more comparable and facilitates interpretation of regression coefficients (Gelman 2008). In addition, continuous variables were categorized into quartiles (each group containing 25% of the data) to facilitate the modeling of nonlinear relationships and mitigate skewness and extreme outliers (Pienkowski et al. 2025), with the first quartile (Q1) set as the reference category. To account for multicollinearity or redundancy among explanatory variables, we performed a variance inflation factor (VIF) analysis prior to fitting the INLA model, considering VIF < 5 as a condition to keep a variable in the model (Silva et al. 2020).

Fixed effects were assigned Gaussian priors with mean 0 and precision 0.001 (variance = 1000), corresponding to weakly informative priors on the logit scale, as implemented by default in R-INLA. The precision parameter (τ) of the municipality-level random intercept (iid specification) was assigned a Gamma prior with shape = 1 and rate = 0.00005 (equivalently, a log-Gamma prior on log precision), following the default INLA parameterization. These weakly informative priors allow substantial variability while preventing overfitting, ensuring that posterior inferences are primarily driven by the data. We then fitted a hierarchical logistic regression model with municipality-level random intercepts, assuming a latent Gaussian structure. INLA approximates the marginal posterior distributions of fixed effects and hyperparameters using Laplace approximations. The complete model specification, including priors, is provided as supplementary data (see Data Availability), which allows full replication of our analysis.

For each covariate, we report the posterior mean, 95% credible intervals (CrI), and interpret associations as statistically meaningful when the interval does not include zero on the logit scale (or one on the odds-ratio scale). Inference was based on posterior marginal distributions of fixed effects and hyperparameters, as computed via the INLA.

### Predictive scenario and model validation

We used the posterior participation probabilities from the Bayesian INLA model to identify potential new candidates for the PCMA among non-participating properties. The optimal classification cutoff was defined using the ROC-based Youden index (Unal 2017), ensuring a balanced trade-off between sensitivity and specificity. This predictive application of INLA follows Bayesian decision analysis principles for latent Gaussian models (Rue et al. 2009; Moraga 2019), enabling estimation of likely program expansion and associated budgetary needs based on observed payment data.

Model performance was assessed through a leave-one-municipality-out (LOMO) cross-validation (Roberts et al. 2017), which controls for spatial autocorrelation by ensuring independent training and testing areas (Jain and Zimmerman 2024). Additional bootstrap validation with 100 iterations (Hastie et al. 2009) provided estimates of predictive accuracy and uncertainty. Performance metrics included the area under the ROC curve (AUC) and classification accuracy across all runs. Details on threshold selection, validation workflow, and uncertainty estimation are provided in Supplementary Material—SM Predictive scenario and SM Model validation.

## Results

The analysis identified a total of 18 717 rural properties within the 20 municipalities with 974 PCMA participants. Although 90% of all rural properties (including among PCMA participants) fell within the group of ≤ 4 fiscal modules (i.e., small properties), there was a large size variance among small properties (mean size 21 ha, ± 26 SD). According to our results, the region is greatly impacted by a hilly topography, and with an elevation gradient ranging from 1 to 2397 m (Supplementary Material—SM Fig. S1, and SM Table S1). Average annual precipitation at 1533 mm/year (± 190 SD) was not evenly distributed across the study region as it tended to be concentrated to higher elevation areas (SM Figs. S2 and S3). The study region was also covered by protected areas of different status (e.g., integral protection, sustainable use), and 368 PCMA participants were observed within them (exclusively within sustainable use, Fig. 1). The valley regions are crossed by major national roads (i.e., Regis Bitencourt highway in Ribeira Valley, and Dutra highway in Paraíba Valley). However, because of their valley biophysical configuration (with major roads along lower valley floors leaving highlands and hilly terrain distant) and major presence of small properties, good quality roads are unevenly distributed (SM Fig. S4).

The shape of each violin plot in Supplementary Fig. 1 (SM Fig. S1) represents the kernel density estimation of the distribution of each explanatory variable, where wider sections indicate a higher concentration of rural properties with similar values. For instance, the variable ‘Pasture quality (2017)’ presents a clear bimodal pattern: two wider areas in the violin plot indicating two frequent quality levels among the properties, likely corresponding to low and high pasture quality groups. This suggests that landholders often fall into either degraded or vigorous pasture conditions, with fewer properties in intermediate states. In contrast, ‘Transportation cost (2017)’ displays a wide and relatively symmetrical distribution, with no single dominant value range—indicating that remoteness varies gradually and continuously across the study region.

**Fig. 2:**
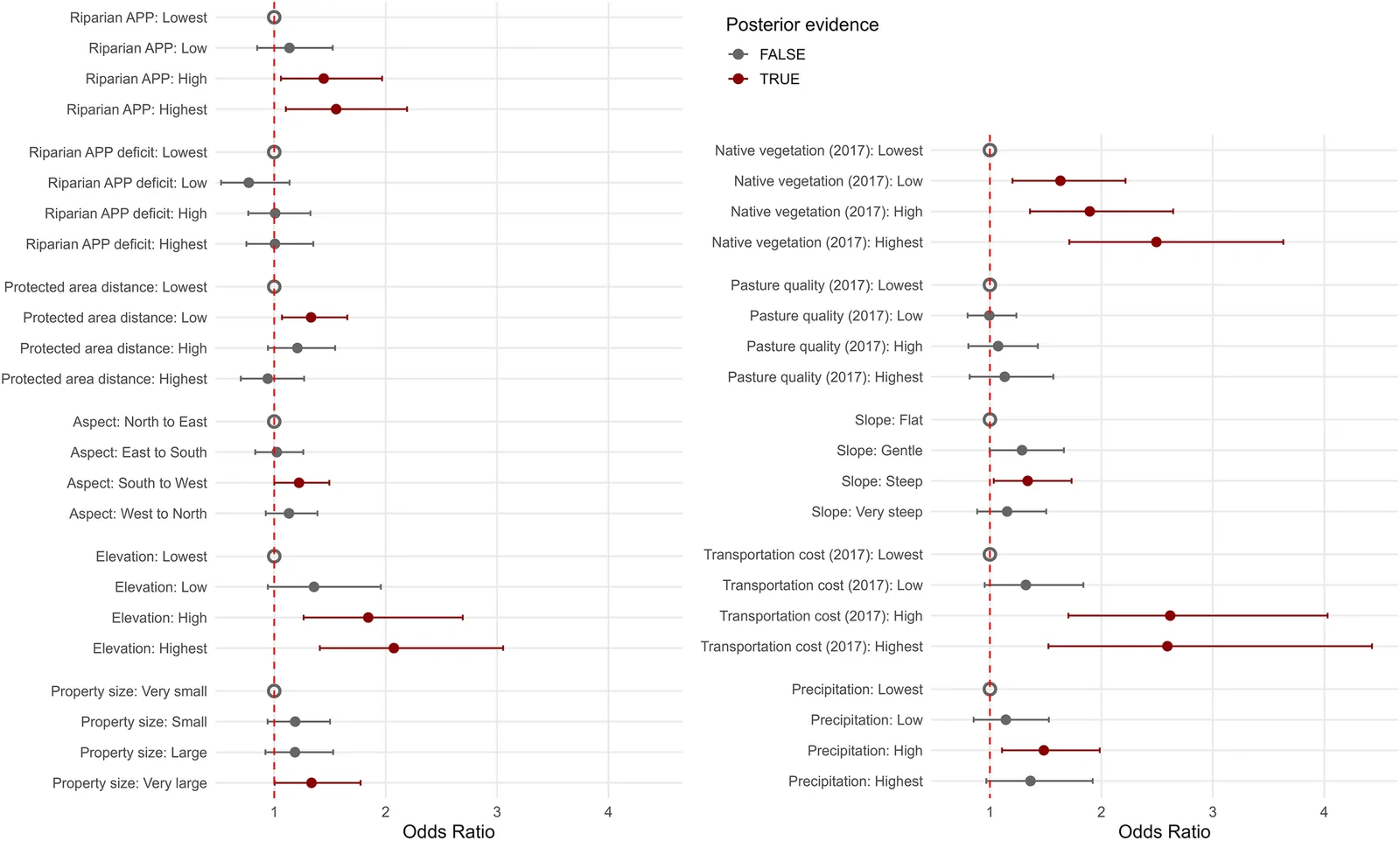
Estimated odds ratios representing the associations between participation in the *Projeto Conexão Mata Atlântica* and explanatory variables across 18 717 rural properties. Hollow markers on the red center line indicate reference categories; solid points show the estimated effects, and whiskers represent 95% CrI (red markers indicate variables for which CrI do not encompass an odds ratio of 1)

The INLA model identified several socioenvironmental predictors with strong posterior evidence of association with landowner participation in the PCMA program (Fig. 2), corroborating several a priori assumptions (Table 1). Statistical importance was assessed through the posterior marginal distributions: predictors whose 95% CrI did not overlap zero (or, equivalently, whose odds-ratio intervals did not include 1) were considered to have meaningful effects. Deficits of native vegetation within riparian APP and pasture quality were the only variables that showed no clear association for all levels (the 95% CrI included 1). In contrast, the percentage of native vegetation in 2017 and riparian APP were the variables that displayed a consistent and gradually increasing effect on the likelihood of participation (Fig. 2). Compared to properties in the lowest vegetation quartile, those in the highest quartile were 149% more likely to participate (OR = 2.49; 95% CrI 1.71–3.63), while properties in the intermediate quartiles also showed elevated odds (Q2: OR = 1.63; 95% CrI 1.20–2.22; Q3: OR = 1.90; 95% CrI 1.36–2.64). Properties in the highest riparian APP quartile had 56% higher odds of participation (OR = 1.56; 95% CrI 1.10–2.19). These results support the interpretation that landowners with larger conservation-priority areas—including riparian forests—were more likely to engage voluntarily in the program.

Posterior summaries for all fixed effects—including posterior means, standard deviations, 95% CrI, and the corresponding odds ratios—are presented in SM Table S2. We also report the posterior marginal distribution of the random-intercept parameter (τ), which captures the variability among municipalities and reflects the hierarchical structure of the model. Its posterior mean, standard deviation, and 2.5%, 50%, and 97.5% quantiles are summarized in SM Table S3, and its full posterior density is shown in Supplementary Materials Figure S5.

Although property size was not a formal criterion for program selection, the results indicated that larger properties had 34% higher odds of engagement—particularly those classified as large (in our case ≥ 4 FM), for which the 95% CrI did not include 1 (OR = 1.34; 95% CrI 1.00–1.78). Proximity to protected areas conferred 33% higher odds of participation (Q2: OR = 1.33; 95% CrI 1.07–1.66). Similarly, the model showed that rural properties located in regions with higher transportation costs—typically more remote areas—were 159% to 161% more likely to engage in the program (Q3: OR = 2.62; 95% CrI 1.70–4.03; Q4: OR = 2.59; 95% CrI 1.53–4.43). Topographic characteristics revealed noteworthy patterns. Properties at elevations ranging from 781 and 929 m had 84% higher odds of participation (OR = 1.84; 95% CrI 1.26–2.69), and those in the highest elevation quartile had 107% higher odds (OR = 2.07; 95% CrI 1.41–3.05). On steep slopes (23.5°–29.3°), properties were more likely to participate (OR = 1.34; 95% CrI 1.03–1.73), consistent with a reduced agricultural suitability in these locations. The importance of agricultural suitability was further reflected by slope aspect, with an important directional effect toward southwest-facing slopes (179°–216°) being 22% more likely to participate (OR = 1.22; 95% CrI 1.00–1.49). Finally, properties with relatively higher rainfall (over 1600 mm/year) had 48% higher odds of participating (OR = 1.48; 95% CrI 1.11–1.99).

Predictive performance through the LOMO approach reached a mean AUC across all municipalities at 0.667 (0.106 SD), with a 95% quantile-based interval of 0.481–0.847, indicating moderate discriminatory ability of the model across different geographic contexts. To assess the statistical variability of the model’s predictive performance, the bootstrap validation with 100 re-samplings obtained a mean AUC of 0.764 (0.002 SD), indicating a good and stable discriminatory ability. The low standard deviation suggests that the model’s performance remained consistent across the bootstrap samples, reinforcing the robustness of the predictive associations identified.

**Fig. 3:**
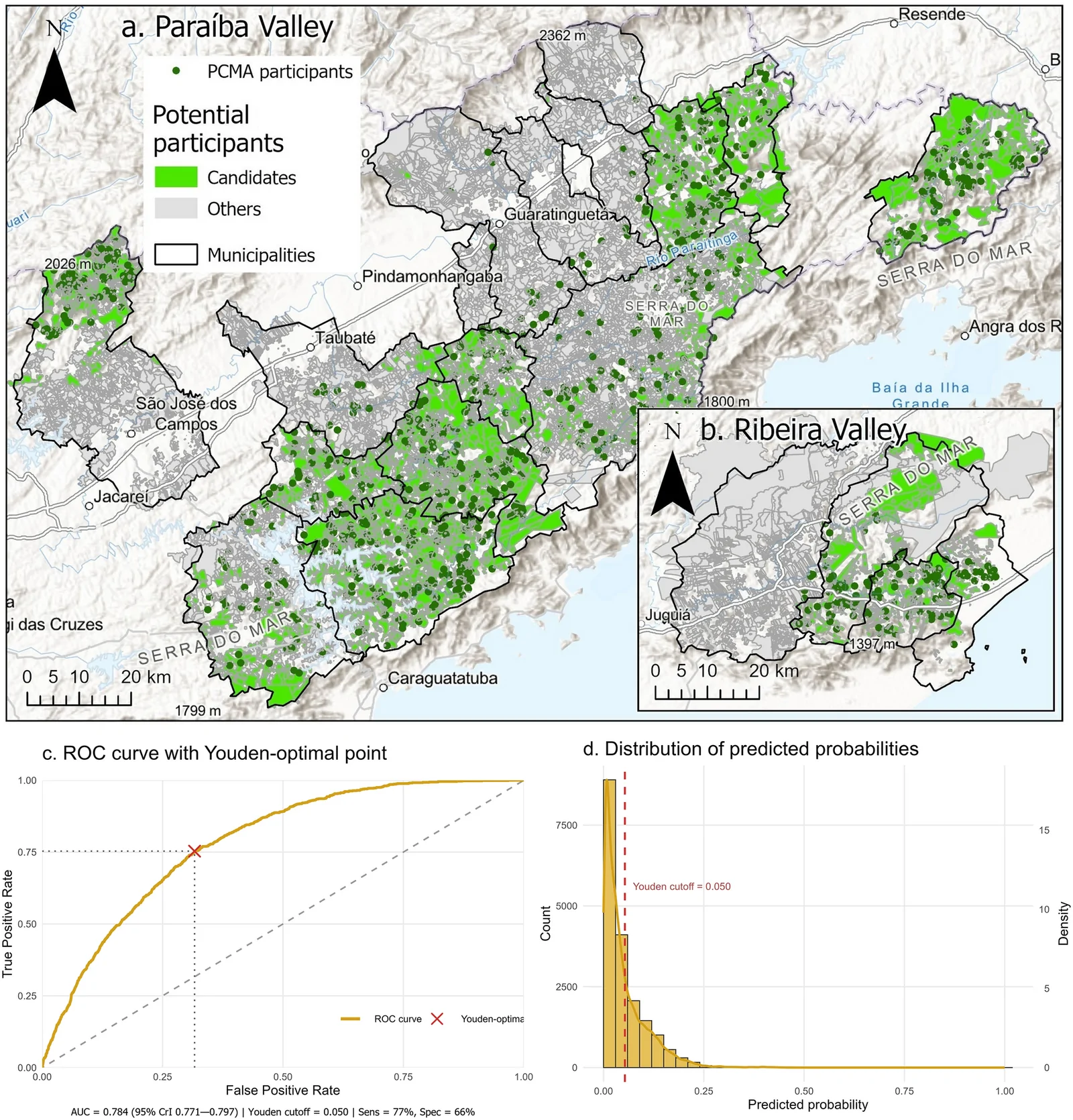
Results of the modeling of participation in the Projeto Conexão Mata Atlântica (PCMA), a conservation program. **a** The Paraíba Valley region with around 800 PCMA participants. **b** The Ribeira Valley, with about 150 PCMA participants. **c** ROC curve of the model highlighting the Youden-optimal cutoff point. **d** Distribution of predicted participation probabilities, with the red dashed line marking the Youden cutoff used to identify properties as additional candidates for participating in the program

The PCMA program had a total cost of R$ 30.3 million (for the 974 participants). Based on the INLA-predicted participation probabilities, we identified 5615 additional rural properties as candidates for expansion of the program (Fig. 3a, b). The ROC analysis indicated good discriminatory ability (AUC = 0.784; 95% CrI 0.771–0.797), with the ROC-optimal Youden index at a cutoff probability of 0.050 (sensitivity ≈ 78%, specificity ≈ 66%; Fig. 3b, c). Using this threshold, we classified non-participants above the cutoff as additional candidates. If all were enrolled, program costs would increase to between R$ 147–206 million, depending on whether mean or median contract costs were applied. This represents a five- to sevenfold budget expansion relative to the current program cost. However, given that voluntary participation does not necessarily align with areas of highest environmental impact, expanding the program without strategic targeting may reduce the environmental returns per unit of investment, something that is particularly important under a limited conservation budget.

## Discussion

Land-use and all related decision-making at the rural property is a reflection of landowners’ viewpoints, perspectives, values, and aspirations (Dou et al. 2023; Fernandes et al. 2025). Given this, it is reasonable to expect that personal, cultural, and contextual factors also influence their decisions to engage with conservation policies (Varella et al. 2025). Our study explored this socioenvironmental dimension by evaluating the participation of rural properties within a conservation program, the *Projeto Conexão Mata Atlântica* (PCMA), in São Paulo state, Brazil. As an incentive “carrot” policy, participation was voluntary and—apart from proximity to protected areas in some contexts (Oliveira et al. 2024)—no social–environmental eligibility conditions were mandated (Silva et al. 2025a). Despite the program’s voluntary design and minimal eligibility criteria, PCMA participation was not spatially random. It clustered across municipalities and properties and varied systematically, with our model attributing part of this heterogeneity to the specified predictors and a municipality-level random intercept.

The results from our INLA model showed that engagement was strongly associated with some socioenvironmental predictors. Topographic factors revealed that participants were more engaged in rural areas with low agricultural capacity. For instance, in the Southern Hemisphere, south-facing slopes receive less solar radiation than their north-facing counterparts, making them less suitable for agricultural production—solar radiation is a key component of agricultural productivity, so less insolated areas are less competitive (Smith and Bookhagen 2021). Consequently, landowners are more likely to abandon or avoid cultivation on these slopes (Silva et al. 2017, 2018), which increases their likelihood for involvement in conservation initiatives. This decision-making is further influenced by the presence of steep slopes, which in the region restricts the use of machinery and other intensive agricultural practices such as high-productivity cattle ranching (Silva et al. 2018). Furthermore, higher elevations are located near the foothills of the *Mantiqueira* (exclusive to the Paraíba Valley) and *Serra do Mar* mountain ranges. These areas are typically remote, difficult to access by roads, and subject to severe agricultural constraints (Silva et al. 2020).

Through the reverse auction method implemented in the PCMA to engage landowners in the ‘protection’ scheme (Silva et al. 2025a), properties with higher proportions of native vegetation became highly incentivized, which increased participation as the proportion on their land increased. This is an expected result and also suggests that landowners who already preserve large parts of their properties as native vegetation, can be rewarded by conservation policies. This may contradict previous findings suggesting that altruistic voluntary conservationists tend to avoid normative contracts and opt out of public conservation programs (Primmer et al. 2014). However, we argue that this ‘rewarding’ framework recognizing the ones who are doing voluntary conservation is an incentive to ensure the long-term maintenance of native vegetation—an outcome attainable if approached by tailored PES schemes (Engel et al. 2008). Financial incentives are therefore not only strategic tools to reward voluntary conservation, but essential mechanisms to lower the initial uptake costs that often prevent landowners from investing in conservation (Selinske et al. 2016; Pereira 2024; Fernandes et al. 2025).

We found larger properties having higher probability of participating in the PCMA. In the Atlantic Forest, ecosystem restoration in larger properties is more likely to occur than in small and medium ones (Pienkowski et al. 2025). Higher compliance demand with the Forest Code and commercial interests were found to be major drivers. In addition, landowners of large properties (although majorly driven by legal compliance) seek to engage with restoration mostly driven by their interests in carbon credit markets—but their individual willingness for restoration does not differ from small/medium landowners (Fernandes et al. 2025). These results are of critical importance because it indicates that landowners of large properties may undertake conservation strategies due the perception of law enforcement, market sanctions, and carbon credits.

Our results also suggest that the concern regarding water resources is a major driver for landowners’ decision to engage in conservation policies. Rural properties with the highest presence (greater amounts) of riparian APPs were the most likely to participate in the PCMA. Although our model did not reach greater ability to predict participation for other regions outside the study area (through the LOMO validation test), this result supports previous findings across the biome indicating water as a major concern underpinning conservation actions or willingness to act (Silva et al. 2018; Fernandes et al. 2025; Pienkowski et al. 2025).

It is important to note that although PCMA was conceived as a federal–state collaboration, each state was responsible for its implementation (e.g., defining how to apply the funds), provided that the overarching goals of native vegetation conservation, water quality, and climate resilience were respected. São Paulo was the only state to implement a PES scheme at a large scale (Silva et al. 2025a), with sophisticated instruments that included heterogeneity in payment schemes, reverse auctions, direct payments, and robust technical support. In contrast, Minas Gerais directed its resources primarily toward restoration activities and sustainable agricultural (e.g., soil recovery, ILPF systems), without establishing a formal PES mechanism. Rio de Janeiro adopted a smaller PES scheme, reaching approximately 200 participants and focusing mainly on technological improvements (MCTI 2024). These differences highlight that the participation patterns we have identified—such as spatial clustering and the influence of riparian APP extent, topography, and vegetation cover—reflect a policy design and operational model unique to São Paulo, rather than a uniform dynamic across states. Altogether, this broader view underscores that conservation programs based on economic incentives depend heavily on institutional design, scale of implementation, and territorial engagement strategies.

### Implications for Atlantic forest conservation

The Atlantic Forest, with around 2.8 million rural properties, has a legal deficit of 4.7 Mha of native vegetation (within ~ 1.6 million properties) that needs restoration to reach full compliance with the Forest Code (Faria et al. 2021). In addition, this biome hosts nearly 65% of the Brazilian population (Costa et al. 2024), up to 70% of the national GDP, and is the only Brazilian biome that has bent the deforestation curve, showing positive net gains of native vegetation since 2006—an indication of a biome-level forest transition (Vancine et al. 2024; Silva et al. 2025b). This scenario suggests strong progress in Atlantic Forest conservation. However, reconciling agricultural practices and other land uses with conservation within working landscapes remains a global challenge (Garibaldi et al. 2020), especially in the Atlantic Forest which lost about 16 Mha of native vegetation in the last 40 years (Silva et al. 2025b) mainly due to agriculture (Silva et al. 2023).

From a local perspective, if a long-term conservation policy is put in place to support the participation of new landowners in the valleys of Paraíba and Ribeira, our model indicates the potential engagement of 5615 additional properties—about 30% of the rural properties in the region—at a cost of R$ 206 million (0.006% of the São Paulo state’s GDP of 2024). Because economic resources for conservation are usually scarce, spatial targeting is key to optimize implementation (Rouveyrol and Prima 2024), as we show.

However, PCMA results indicating self-selection of landowners—pushing conservation policies toward marginal lands while deforestation continues in highly productive areas—reveal a limited reach of such programs and presence of enrollment bias in such program. In this context, the PCMA falls short of addressing a broader environmental governance issue: incentive-based programs that rely solely on voluntary participation tend to consolidate existing conservation behavior rather than transform land-use trajectories, which is necessary to address continued degradation in a highly endangered biome. Similar dynamics have been observed across Latin America where conservation programs had low impact in coping deforestation because of enrollment bias toward selecting properties with reduced deforestation risk (Robalino and Pfaff 2013). Addressing this limitation requires integrating economic instruments with territorial planning, regulation, and social innovation to foster genuinely inclusive and scalable conservation transitions.

While previous studies have pointed to the need to target landowners in marginal lands to enhance participation and policy effectiveness (Viani et al. 2019), we argue that conservation programs aiming to reconcile agriculture, conservation, and restoration in highly threatened tropical ecosystems such as in the Atlantic Forest must transcend the enrollment bias (Robalino and Pfaff 2013) and effectively reach productive, highly (e.g., economically) integrated lands.

A remaining challenge is whether the provision of ecosystem services is sustained after programs end (e.g., with the completion of payments), and whether payments under PES schemes are competitive with opportunity costs (Salzman et al. 2018). Thus, conservation programs must pay attention to the factors that strengthen or weaken autonomous motivations and recognize local contexts that facilitate the continuity of conservation actions (Le et al. 2024). In addition, conservation programs must consider the strategy and tools to be applied (e.g., which PES scheme), whether based on incentives or rewards, since each model establishes different dynamics of engagement. In the PCMA’s incentive-based model, the logic of reciprocity embedded in financial transfers may contribute to building and reflecting social bonds, playing a role in the recognition and delivery of conservation actions (Vatn 2010).

## Conclusion

Results of this study highlight that engagement decisions are not random. Our analysis provides direct answers to the research questions posited in the Introduction. To our first question (i) we found that participation was strongly associated with topography, vegetation cover, riparian APP extent, transportation costs, distance from protected areas, precipitation, and rural property size, while some predictors—such as pasture quality and riparian vegetation deficit—showed no meaningful effect. To our second question (ii) we found that the PCMA has been effective at retaining landowners already ‘engaged’ in conservation on marginal lands within a working landscape, but less successful at engaging those with more productive agricultural lands. These findings illustrate that voluntary conservation programs often reinforce existing patterns of engagement rather than expanding participation among more production-oriented landowners—i.e., enrollment bias. Beyond the PCMA case, this highlights a broader global challenge: incentive-based conservation tends to succeed where opportunity costs are low, but struggles to compete with profitable land uses. Designing policies that overcome these structural asymmetries requires context-specific incentives, adaptive governance, and spatial targeting capable of balancing equity, efficiency, and ecological outcomes in human-dominated working landscapes worldwide.

## Supplementary Information

Below is the link to the electronic supplementary material.

Supplementary file1 (PDF 1226 KB)

## Data Availability

Anonymized and non-spatially explicit data, together with the R script used in the study, are available from: https://github.com/laecas/INLA-SocioEnvironmental-Participation. Links to original data sources are provided in the Supplementary Material, section SM Datasets.
