# Supplementary material for: Who joins voluntary conservation programs? Socioenvironmental predictors of participation in tropical working landscapes
Source: Ambio. 2026 Apr 4;55(10):2466–79. doi: 10.1007/s13280-026-02381-3 (PMC13554016; doi:10.1007/s13280-026-02381-3)
Supplement: Supplementary file 1 [file 13280_2026_2381_MOESM1_ESM.pdf]

Supplementary Material

Who Joins Voluntary Conservation Programs? Socioenvironmental Predictors of Participation in Tropical Working Landscapes

SM Datasets

This study relies on a series of datasets from publicly available sources, except the data on private rural properties participating on the PCMA, which is only available under request to the SEMIL. Native vegetation was derived from the MapBiomas (MapBiomas, 2023a), with "Forest" and "Non-forest natural formation" classes combined, from the 'land-cover' product version 9. Information on pasture quality, which indicates the level of its degradation or vigor, was also obtained from MapBiomas (MapBiomas, 2023b), 'pasture vigor' product, version 2. All MapBiomas data are provided at 30-meters spatial resolution (raster format). Topography was obtained from the INPE/TOPODATA elevation product (http://www.dsr.inpe.br/topodata/index.php), available at 30-meters resolution (Valeriano, 2008). Precipitation data were obtained from the WorldClim version 2.1, at 1-km resolution (https://www.worldclim.org/). Rural property information and areas defined for adequacy by the national Forest Code were obtained from the *Rural Environmental Cadastre* (CAR; https://www.car.gov.br/#/) system, in shapefile format (Silva et al., 2025). Also in shapefile format, the legal Protected Areas were obtained from the *Environmental Spatial Data Infrastructure of São Paulo State* (DataGEO system; https://datageo.ambiente.sp.gov.br/). Transportation cost was derived from the Brazilian 'Transportation Costs' dataset developed by Victoria et al. (2021), provided in raster format at 550-m resolution.

SM Data preparation

To enable spatially explicit modeling, all datasets were harmonized at the rural property level. Spatial zonal statistics techniques were applied to each raster dataset (Silva et al., 2023), allowing the aggregation of pixel-level information into property-level metrics. Native vegetation was calculated as the percentage of each rural

property covered by native vegetation in 2017 (the year immediately preceding the implementation of the PCMA) and represents the previous status of the property. Pasture quality, originally classified into three categories (1-*low*, 2-*medium*, 3-*high*), was summarized by computing the mean value across each property for 2017. Climatic and transportation cost variables were also aggregated using property-level averages. Annual precipitation (mm/year) corresponds to the 30-year mean (1970—2000) provided by WorldClim. Transportation cost was derived for the year 2017 and expressed as country-wide index ranging from 0 to 52,000, with higher values indicating greater transportation constraints.

Topographic variables were extracted from the TOPODATA elevation product. From this dataset, elevation (m), slope (degrees), and aspect were derived and averaged for each property. According to the Forest Code, slopes ≥ 45° are considered Permanent Preservation Areas (APP) while between 25° and 45° have restricted uses. Additionally, hilltops and elevations above 1800 meters are also designated as APPs. From the CAR data, our model used two variables. The number of fiscal modules (FM, i.e., the minimum economically viable property size—properties ≤ 4 FM are small, according to the Agrarian Reform Law 8629 of 1993), which indicates the rural property size), and the riparian APPs. Based on the riparian APP layer, we computed the native vegetation deficit within them, expressed as the percentage of area lacking native vegetation in each property. According to the Forest Code, riparian APPs include buffer zones along riverbanks ranging from 30 to 500 meters, depending on the river's width, as well as a 50-meter radius buffer around springs—therefore a proxy to presence of water resources within the property. The FM size varies by municipality, and for this study region the average of one module is 20 ha (Silva et al., 2025). For protected areas, we calculated the mean Euclidean distance from each property to the nearest protected area boundary, using a 30-meter resolution raster generated within the study area. Properties located entirely within protected areas were assigned a distance value of zero. Due to complex polygon shapes and sizes, in our analysis we excluded the properties under the CAR data smaller or equal to 0.5 ha to avoid artifacts, as suggested by Pienkowski et al. (2025).

SM Predictive scenario

To explore the potential expansion of the PCMA, we used the participation probabilities estimated by the Bayesian INLA model. To this effect, the model provides the probability of participation for each rural property given its socio-environmental characteristics. To identify potential new candidates among non-participants, we applied the Receiver Operating Characteristic (ROC)-optimal Youden threshold, a widely used method for selecting the classification cutoff that maximizes the balance between sensitivity (correctly identifying participants) and specificity (avoiding false positives) (Unal, 2017). In practice, the Youden index corresponds to the maximum vertical distance between the ROC curve and the diagonal line of a random classification, ensuring the best trade-off between true and false classifications. This counterfactual classification allows estimating where program expansion is most likely and to calculate the budgetary implications of enrolling additional candidates. Such predictive use of INLA is consistent with Bayesian decision analysis frameworks applied to latent Gaussian models (Rue et al., 2009), which have shown to be useful for policy-relevant analyses (Moraga, 2019).

To approximate costs, we used the observed total payments per contract as the empirical basis—information that is provided along with the PCMA dataset. The mean and median costs per existing participant were applied to candidate properties, to project total program costs under a scenario of full expansion. This approach reflects the actual expenditure structure of the program, avoiding assumptions that costs scale directly with total farm area.

SM Model validation

Due to the small number of municipalities, to evaluate the predictive performance of the model in a spatially robust manner, we implemented a leave-one-municipality-out (LOMO) cross-validation. The LOMO approach follows the principles of spatial block cross-validation as recommended by Roberts et al. (2017), and helps controlling for spatial autocorrelation, while improving model generalizability by ensuring that

training and testing data are spatially independent (Jain and Zimmerman, 2024). Hence, each of the 20 municipalities was sequentially withheld as the validation set, while the model was trained on the remaining 19. Predictions were made for the withheld municipality, and performance was summarized using the Area Under the ROC Curve (AUC) and classification accuracy across all iterations. This approach accounts for spatial heterogeneity and avoids information leakage among spatial units. In addition, this is a useful validation procedure for the purposes of spatial generalization to other contexts (e.g., municipalities outside the study region), as it captures regional heterogeneity.

To assess the statistical variability of model performance, we also validated the model using a bootstrap procedure with 100 iterations (Hastie et al., 2009). In each iteration, a random sample comprising 70% of the data (with replacement) was used for model training, and predictions were made for the remaining 30% (out-of-bag data). The AUC and classification accuracy were used to evaluate model performance on the test set across iterations. This approach provides a robust estimate of the model's average predictive accuracy and its variability under repeated sampling. Predictions for the test data were obtained simultaneously with model fitting, ensuring consistency in the uncertainty estimation. This procedure follows established statistical practices for model validation, where bootstrap resampling is widely used to assess predictive accuracy and variability (Hastie et al., 2009) and is consistent with recommendations for robust model evaluation in ecological applications (Zurell et al., 2016).

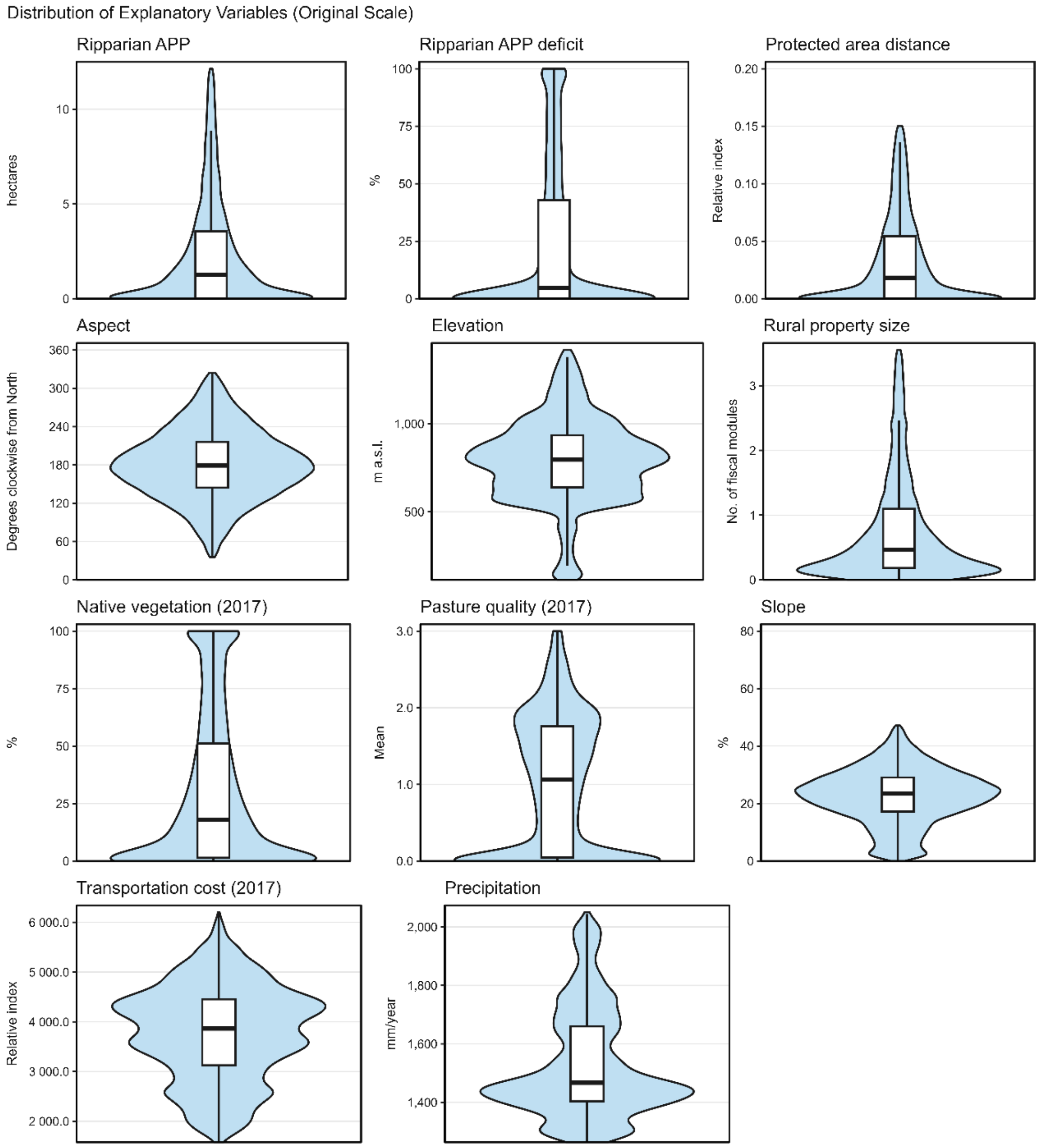

SM FIGURE S1. Violin plots displaying the distribution of explanatory variables used in the analysis of 18,717 rural properties. Each panel shows a different variable, with the width of the violin indicating the density of observations. Boxplots within each violin indicate the interquartile range and median values. Outliers were excluded for APP and rural property size to improve visualization.

Table S1. Descriptive statistics of all explanatory variables used in the analysis. Values are reported as mean, standard deviation (SD), median, first (Q1) and third (Q3) quartiles, interquartile range (IQR), minimum and maximum.

Variable	Unit	Mean	SD	Median	Q1	Q3	IQR	Min	Max
APP	hectares	5,626	22,427	1,584	0	4,854	4,854	0	1266,659
APP deficit	%	24,281	32,299	4,672	0	42,814	42,814	0	100
Aspect	Degrees clockwise from North	180,159	55,863	178,866	143,833	216,03	72,198	0	353,322
Elevation	m a.s.l.	751,8	308,568	780,745	602,958	929,19	326,232	0	1851,998
Native vegetation (2017)	%	30,528	32,79	17,992	1,401	51,138	49,737	0	100
Pasture quality (2017)	Mean	1,03	0,861	1,062	0,046	1,757	1,711	0	3
Precipitation	mm/year	1533,616	187,589	1467,645	1403,524	1662,466	258,942	1263,655	2311,955
Protected area distance	Relative index	0,039	0,046	0,021	0	0,06	0,06	0	0,205
Rural property size	No. of fiscal modules	2,394	28,443	0,569	0,204	1,544	1,34	0	2653,913
Slope	%	23,038	9,857	23,533	17,314	29,254	11,94	0	74,175
Transportation cost (2017)	Relative index	3778,682	946,629	3864,622	3124,042	4453,761	1329,719	1579,33	6202,591

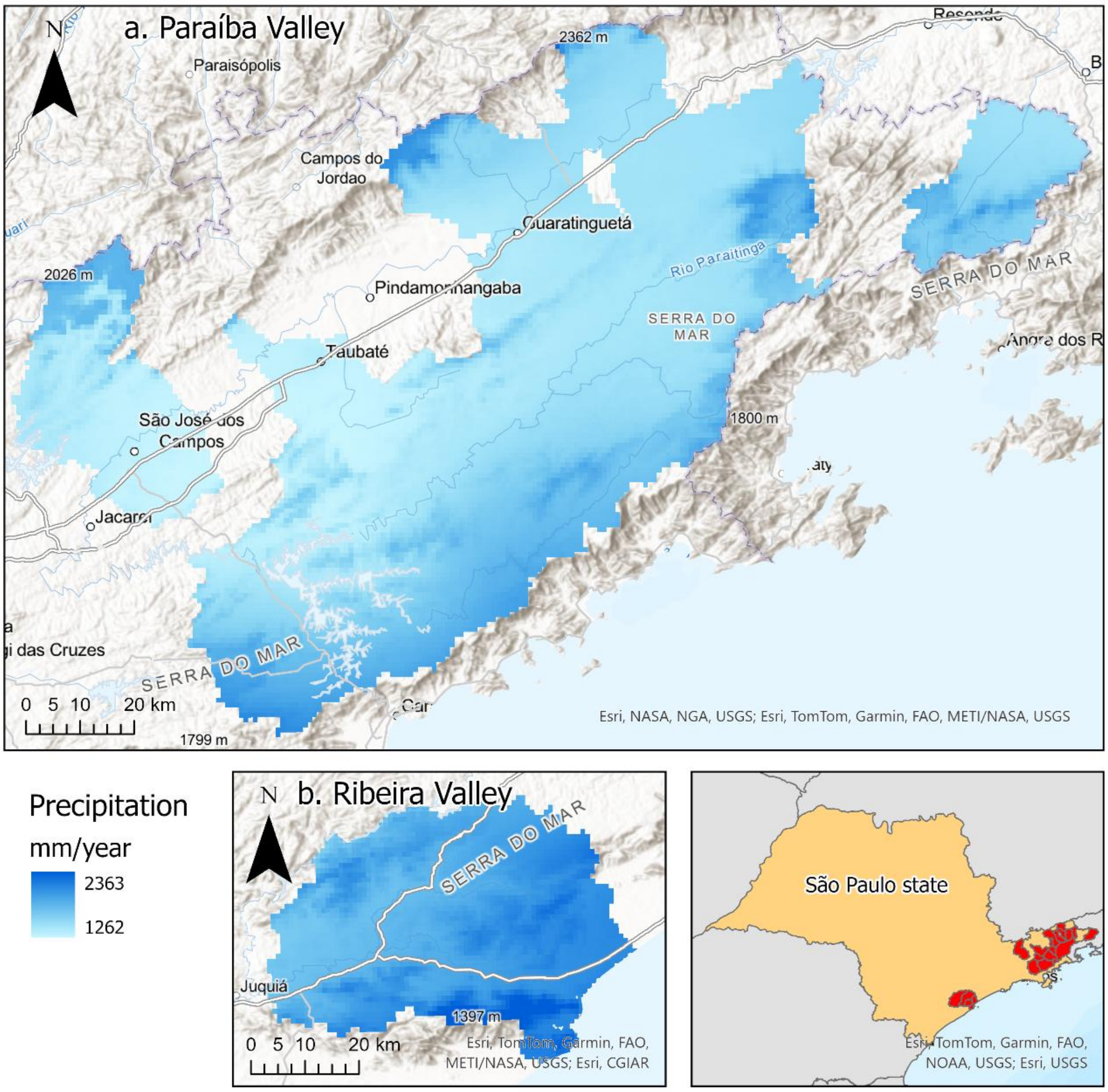

SM FIGURE S2. São Paulo state's areas targeted by the Projeto Conexão Mata Atlântica (PCMA), a conservation program. The figure highlights the precipitation pattern of the areas according to the WorldClim dataset. **a**, The Paraíba Valley. **b**, The Ribeira Valley.

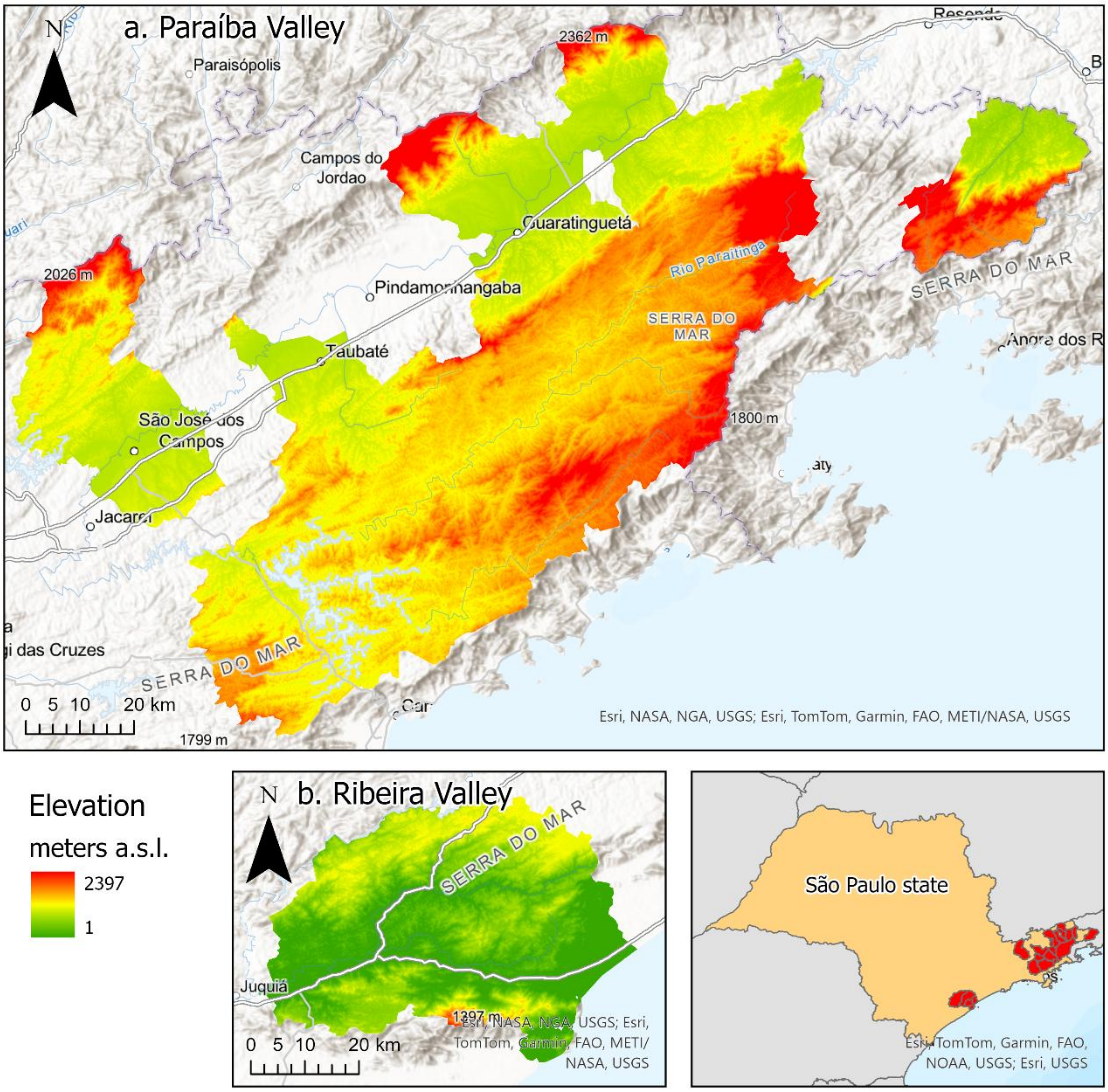

SM FIGURE S3. São Paulo state's areas targeted by the Projeto Conexão Mata Atlântica (PCMA), a conservation program. The figure highlights the elevation pattern of the areas according to the TOPODATA dataset. **a**, The Paraíba Valley. **b**, The Ribeira Valley.

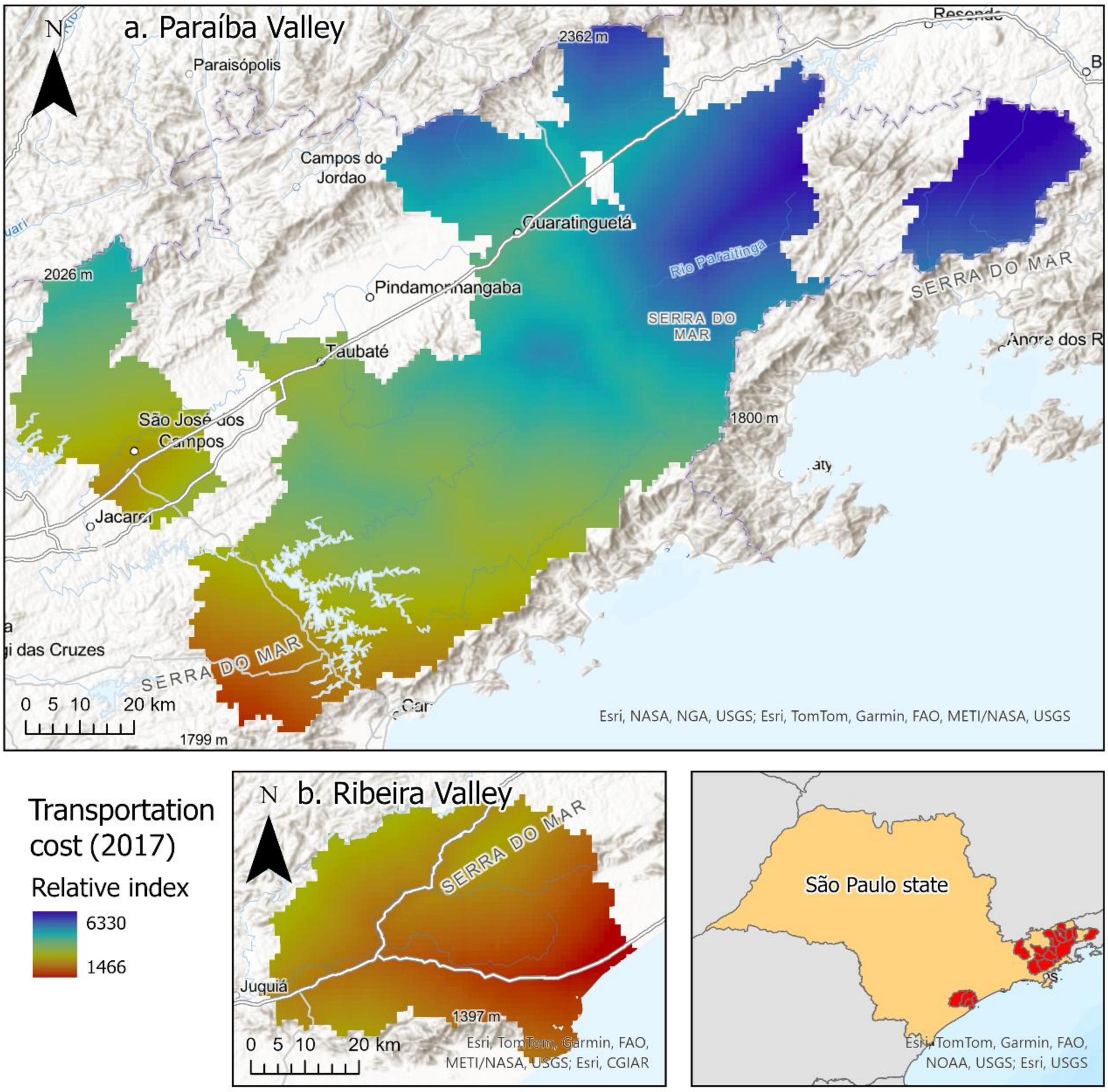

SM FIGURE S4. São Paulo state's areas targeted by the Projeto Conexão Mata Atlântica (PCMA), a conservation program. The figure highlights the transportation costs pattern of the areas according to Victoria's et al. (2021) "transportation cost map" of Brazil. **a**, The Paraíba Valley. **b**, The Ribeira Valley.

Table S2. Summary of the posterior distributions of the parameters.

Variable	Parameter	Quartile	mean	sd	q025	q50	q975	OR	CrI_lower	CrI_upper	Posterior evidence
(Intercept)	(Intercept)	NA	-5.85697	0.419362	-6.69651	-5.8518	-5.04717	0.002859907	0.001235211	0.00642747	TRUE
APP deficit: Low	(2.81e-07, 4.67]	Q2	-0.25877	0.197818	-0.64667	-0.25877	0.129131	0.772001153	0.523789733	1.137835869	FALSE
APP deficit: High	(4.67, 42.8]	Q3	0.008608	0.139319	-0.26458	0.008608	0.281801	1.008647799	0.767530964	1.325512203	FALSE
APP deficit: Highest	(42.8, 100]	Q4	0.006821	0.149963	-0.28724	0.006821	0.300885	1.006848453	0.750336823	1.351053723	FALSE
APP: Low	(3.87e-07, 1.58]	Q2	0.128297	0.149957	-0.16576	0.128297	0.422346	1.136883089	0.847251466	1.525519851	FALSE
APP: High	(1.58, 4.85]	Q3	0.36784	0.157766	0.058475	0.36784	0.677203	1.444604431	1.060218852	1.968346556	TRUE
APP: Highest	(4.85, 1.27e+03]	Q4	0.442267	0.174757	0.099583	0.442267	0.784948	1.556225938	1.104713233	2.192273761	TRUE
Aspect: West to North	(216, 353]	Q4	0.124634	0.10377	-0.07885	0.124633	0.328116	1.132733797	0.924183094	1.388346712	FALSE
Aspect: East to South	(144, 179]	Q2	0.023046	0.106681	-0.18614	0.023045	0.232238	1.023315282	0.830158244	1.261417904	FALSE
Aspect: South to West	(179, 216]	Q3	0.201212	0.102399	0.000419	0.201212	0.402006	1.222885439	1.000422929	1.494819081	TRUE
Elevation: Low	(603, 781]	Q2	0.305556	0.186364	-0.05946	0.305403	0.671443	1.35743474	0.942319936	1.957130077	FALSE
Elevation: High	(781, 929]	Q3	0.611878	0.192762	0.234215	0.611757	0.990226	1.843937393	1.26395129	2.69190908	TRUE
Elevation: Highest	(929, 1.85e+03]	Q4	0.729439	0.197186	0.343262	0.729262	1.11662	2.073993804	1.40959426	3.054622095	TRUE
Farm size: Small	(0.204, 0.568]	Q2	0.172787	0.119115	-0.06079	0.172787	0.406359	1.188611158	0.9410253	1.501334776	FALSE
Farm size: Large	(0.568, 1.54]	Q3	0.171266	0.129371	-0.08242	0.171267	0.424949	1.18680504	0.920886936	1.529505733	FALSE
Farm size: Very large	(1.54, 2.65e+03]	Q4	0.288934	0.145317	0.003981	0.288934	0.573887	1.335004186	1.003992427	1.775148634	TRUE
Native vegetation (2017): Low	(1.4, 18]	Q2	0.490241	0.155929	0.184478	0.490242	0.796001	1.632690113	1.202584089	2.216615571	TRUE
Native vegetation (2017): High	(18, 51.1]	Q3	0.63971	0.169667	0.307007	0.63971	0.972409	1.895899629	1.359337516	2.64424486	TRUE
Native vegetation (2017): Highest	(51, 1.100]	Q4	0.913802	0.191741	0.537815	0.913803	1.289784	2.493739585	1.712240246	3.631904905	TRUE
Pasture quality (2017): Low	(0.0459, 1.06]	Q2	-0.00503	0.111292	-0.22324	-0.00504	0.213226	0.99499257	0.799936295	1.237678435	FALSE
Pasture quality (2017): High	(1.06, 1.76]	Q3	0.0715	0.146053	-0.21488	0.071495	0.357913	1.07413033	0.80664982	1.430353424	FALSE
Pasture quality (2017): Highest	(1.76.3]	Q4	0.124881	0.16603	-0.20068	0.124878	0.450461	1.133023137	0.818186142	1.569042094	FALSE
Precipitation: Low	(1.4e+03, 1.47e+03]	Q2	0.133857	0.148386	-0.15713	0.133863	0.42481	1.143213177	0.854581475	1.52927317	FALSE
Precipitation: High	(1.47e+03, 1.66e+03]	Q3	0.394544	0.148628	0.103055	0.394559	0.685947	1.483683971	1.108538413	1.985614718	TRUE
Precipitation: Highest	(1.66e+03, .2.31e+03]	Q4	0.310624	0.174916	-0.03246	0.310656	0.653524	1.364237883	0.968033434	1.922243599	FALSE
Protected area distance: Low	(2.45e-07, 0.0215]	Q2	0.285826	0.111548	0.067115	0.285817	0.504586	1.330873445	1.069431674	1.656311955	TRUE

Protected area distance: High	(0.0215.0, 0601]	Q3	0.189103	0.126168	-0.05827	0.189092	0.436538	1.20817657	0.943406836	1.547352599	FALSE
Protected area distance: Highest	(0.0601, 0.205]	Q4	-0.05922	0.151602	-0.35643	-0.05925	0.238123	0.942512806	0.700182799	1.268885272	FALSE
Slope: Gentle	(17.3, 23.5]	Q2	0.253463	0.130504	-0.00244	0.253463	0.509368	1.288473422	0.997558604	1.664222657	FALSE
Slope: Steep	(23.5, 29.3]	Q3	0.291937	0.131487	0.034102	0.291937	0.54977	1.33901491	1.034692588	1.732841508	TRUE
Slope: Very steep	(29.3, 74.2]	Q4	0.144023	0.135141	-0.12098	0.144024	0.409019	1.154903379	0.886052713	1.505323643	FALSE
Transportation cost (2017): Low	(3.12e+03, 3.86e+03]	Q2	0.27989	0.167645	-0.04864	0.279817	0.608837	1.323032096	0.952560495	1.83835866	FALSE
Transportation cost (2017): High	(3.86e+03.4, 45e+03]	Q3	0.962181	0.21944	0.533113	0.961751	1.393691	2.617688566	1.704428937	4.030113715	TRUE
Transportation cost (2017): Highest	(4.45e+03.6, 2e+03]	Q4	0.952524	0.271788	0.422044	0.951657	1.487915	2.592623705	1.525304613	4.428475201	TRUE

Table S3. Summary of the posterior values of the hyperparameter.

Hyperparameter	mean	sd	q025	q050	q975	mode
Precision for municipality random intercept (τ)	0.7034	0.2579	0.3098	0.6680	1.3085	0.5997

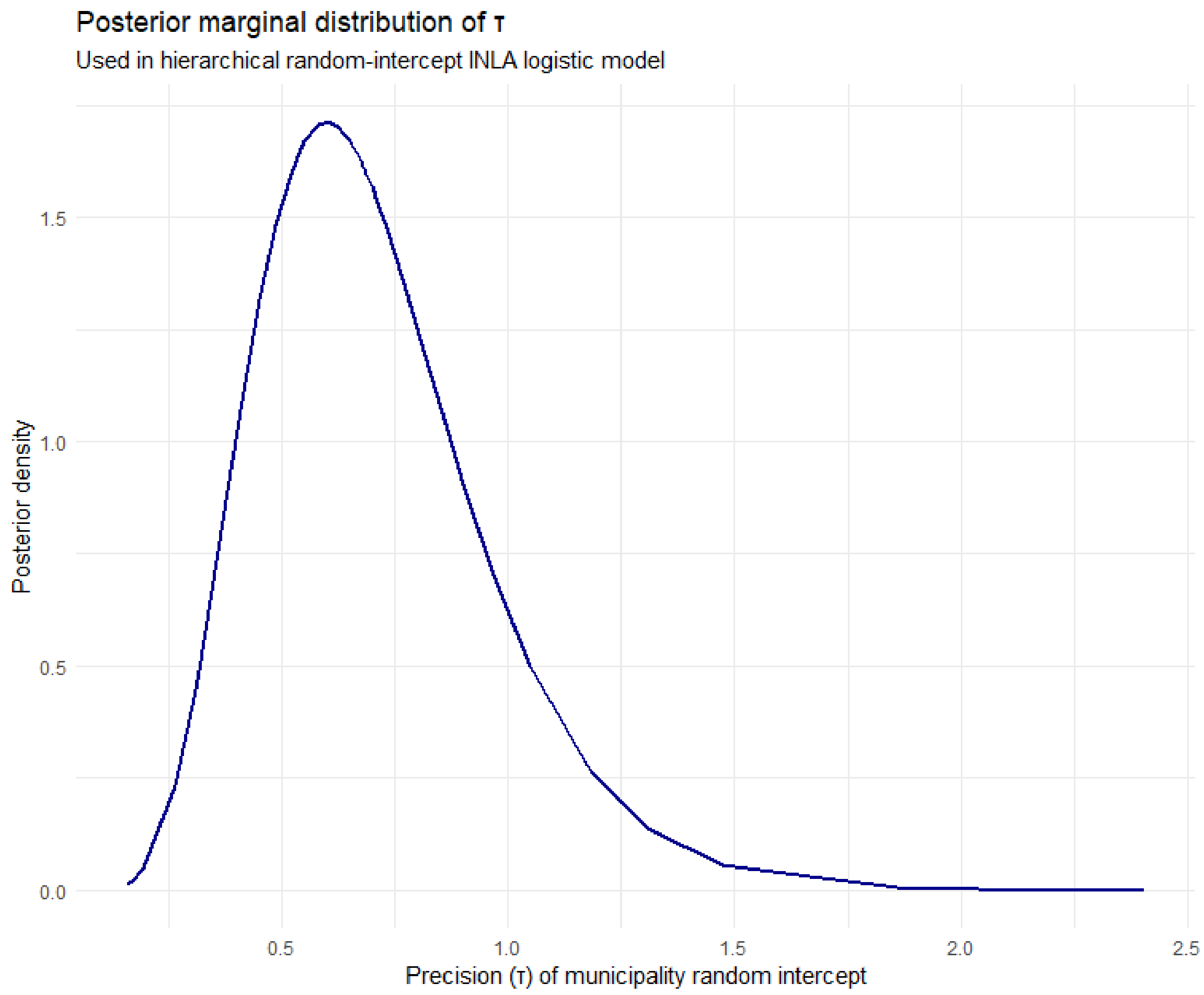

SM FIGURE S5. Posterior marginal distribution of the precision (τ; mean = 0.70; 95% CrI = 0.31–1.31) of the municipality-level random intercept in the hierarchical INLA logistic model. Higher values of τ indicate lower variability among municipalities, whereas lower values reflect stronger between-municipality heterogeneity.

References

1. MapBiomas. (2023a). Projeto MapBiomas – Coleção versão 9.0 da Série Anual de Mapas de Uso & Cobertura da Terra do Brasil. https://mapbiomas.org/
2. MapBiomas. (2023b). Projeto MapBiomas – Coleção versão 9.0 da Série Anual da condição de vigor da pastagem no Brasil. https://plataforma.brasil.mapbiomas.org/
3. Pienkowski, T., Sterrantino, A. F., Tedesco, A. M., Clark, M., S. Brancalion, P. H. S., Jagadish, A., Mendes, A., Siqueira, L. P., & Mills, M. (2025). Spatial predictors of landowners' engagement in the restoration of the Brazilian Atlantic Forest. *People and Nature*, *7*, 247–264. https://doi.org/10.1002/pan3.10765
4. Silva, R. F. B., Millington, J. D. A., Viña, A., Dou, Y., Moran, E. F., Batistella, M., Lapola, D. M., & Liu, J. (2023). Balancing food production with climate change mitigation and biodiversity conservation in the Brazilian Amazon. *Science of The Total Environment*, *904*, 166681. https://doi.org/10.1016/j.scitotenv.2023.166681
5. Silva, R. F. B., Altivo, F., Millington, J. D. A., Dou, Y., Viña, A., Ribeiro, M. C., Vieira, S. A., & Liu, J. (2025). Positive effects of an Atlantic Forest program of payment for ecosystem services on native vegetation and pasture quality. *Perspectives in Ecology and Conservation*, *23*(3), 191–199. https://doi.org/10.1016/j.pecon.2025.06.005
6. Valeriano, M. M. (2008). *TOPODATA: Guia para utilização de dados geomorfológicos locais.* São José dos Campos: INPE. http://urlib.net/ibi/8JMKD3MGP8W/33EPEBL
7. Victoria, D. C., Silva, R. F. B., Millington, J. D., Katerinchuk, V., & Batistella, M. (2021). Transport cost to port though Brazilian federal roads network: Dataset for years 2000, 2005, 2010 and 2017. *Data in Brief*, *36*, 107070. https://doi.org/10.1016/j.dib.2021.107070
